# Synergistic Effect of Co–Ni Hybrid Phosphide Nanocages for Ultrahigh Capacity Fast Energy Storage

**DOI:** 10.1002/advs.201802005

**Published:** 2019-02-20

**Authors:** Zibin Liang, Chong Qu, Wenyang Zhou, Ruo Zhao, Hao Zhang, Bingjun Zhu, Wenhan Guo, Wei Meng, Yingxiao Wu, Waseem Aftab, Qian Wang, Ruqiang Zou

**Affiliations:** ^1^ Beijing Key Laboratory for Theory and Technology of Advanced Battery Materials Department of Materials Science and Engineering College of Engineering Peking University Beijing 100871 P. R. China

**Keywords:** fast energy storage, hollow structure, metal–organic frameworks, phosphide, synergistic effect

## Abstract

Rational design of metal compounds in terms of the structure/morphology and chemical composition is essential to achieve desirable electrochemical performances for fast energy storage because of the synergistic effect between different elements and the structure effect. Here, an approach is presented to facilely fabricate mixed‐metal compounds including hydroxides, phosphides, sulfides, oxides, and selenides with well‐defined hollow nanocage structure using metal–organic framework nanocrystals as sacrificial precursors. Among the as‐synthesized samples, the porous nanocage structure, synergistic effect of mixed metals, and unique phosphide composition endow nickel cobalt bimetallic phosphide (NiCo‐P) nanocages with outstanding performance as a battery‐type Faradaic electrode material for fast energy storage, with ultrahigh specific capacity of 894 C g^−1^ at 1 A g^−1^ and excellent rate capability, surpassing most of the reported metal compounds. Control experiments and theoretical calculations based on density functional theory reveal that the synergistic effect between Ni and Co in NiCo‐P can greatly increase the OH^−^ adsorption energy, while the hollow porous structure facilitates the fast mass/electron transport. The presented work not only provides a promising electrode material for fast energy storage, but also opens a new route toward structural and compositional design of electrode materials for energy storage and conversion.

## 1 Introduction

Energy consumption that relies heavily on the combustion of nonrenewable fossil fuels has caused severe environmental issues in recent years.^1^, ^2^, ^3^, ^4^ Electrochemical energy storage and conversion devices with high energy and power densities as well as long cycling life are highly demanded in order to alleviate the dependence on fossil fuels, and power our society in a more environmentally friendly way.^5^ Many electrochemical energy storage and conversion devices including fuel cells, electrochemical capacitors, and batteries have been intensively investigated and great progress has been made recently.^6^, ^7^, ^8^, ^9^

Among these electrochemical devices, hybrid supercapacitors with fast energy storage capability have attracted increasing attention and showed great promises for fast energy storage due to their higher energy density than common electrical double‐layer capacitors and higher power density than conventional rechargeable batteries.^10^, ^11^, ^12^ Typically, a hybrid supercapacitor is composed of a capacitor‐type electrode as the power source and a battery‐type Faradaic electrode as the energy source.^11^ Carbon‐based materials (e.g., activated carbon and graphene) are usually used for the capacitor‐type electrode while intercalation compounds and transition metal compounds are commonly used as the battery‐type Faradaic electrode materials. For battery‐type Faradaic electrode, transition metal hydroxides have drawn great attention due to their high capacities and low cost, but their irreversible phase transition, large volume change, and poor electrical conductivity result in poor stability and low rate capability during long‐term charge–discharge process.^13^, ^14^ From this perspective, other transition metal compounds that have desirable electrical conductivity (e.g., metal sulfides and selenides) have been recently investigated as potential alternative battery‐type Faradaic electrode materials, which showed superior performance.^15^, ^16^, ^17^ Transition metal phosphides (TMP), especially metal‐rich TMPs (e.g., M_3_P and M_2_P, M = metal), have been proven to have excellent conductivity comparable to pure metal. Although great efforts have been devoted to developing TMPs for electrocatalysis such as hydrogen evolution reaction and oxygen evolution reaction, reports focusing on the utilization of TMPs as battery‐type Faradaic electrode materials are still limited.^18^, ^19^, ^20^, ^21^, ^22^ Meanwhile, rational design of TMPs in terms of structure/morphology and chemical composition of TMPs for hybrid supercapacitors has been rarely reported.^22^

**Scheme 1:**
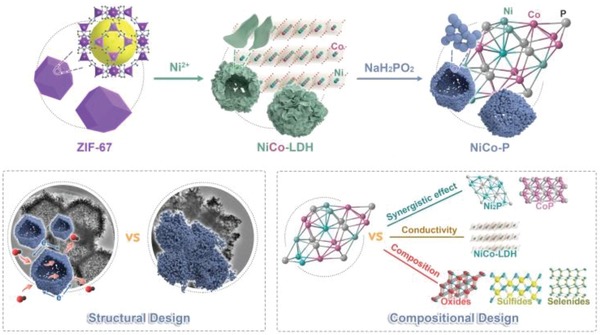
Schematic illustration of the synthetic process of NiCo‐P and the structural and compositional design of NiCo‐P that boost the electrochemical performance for fast energy storage.

Herein, we reported on the structural and compositional design of nickel cobalt bimetallic phosphide (NiCo‐P) nanocages using metal–organic frameworks (MOFs) nanocrystals as sacrificial precursors (**Scheme** 1). From the point of structure, the utilization of the Co‐based MOFs nanocrystals as precursors led to the formation of the well‐defined hollow nanocage structure that can provide abundant redox active sites and facilitate the ion diffusion and charge transport. For compositional design, the Co‐based MOF can serve as Co source and lead to the in situ Co cation substitution/doping into the NiCo‐P, which can induce the synergistic effect between Co and Ni and change the electronic structure of the metal sites, leading to improved OH^−^ adsorption capability for fast energy storage. Moreover, a series of metal compounds including oxides, sulfides, and selenides with hollow structure can also be fabricated using the presented MOF‐templated method. When employed as a battery‐type Faradaic electrode material, NiCo‐P showed excellent electrochemical performance with ultrahigh specific capacity of 894 C g^−1^ at 1 A g^−1^ and high rate capability with capacity retention of ≈72.4% at high current density of 40 A g^−1^. Moreover, a hybrid supercapacitor based on the as‐prepared NiCo‐P and polyaniline/reduced graphene oxide (PANI/rGO) was constructed for more practical application, which showed high energy density of ≈43.4 Wh kg^−1^ at a power density of 620 W kg^−1^, along with good cycling durability with 80.4% capacitance retention after 10 000 cycles at 10 A g^−1^. The excellent electrochemical performance was attributed to the advantageous structural and chemical compositional design of NiCo‐P that led to improved electrical conductivity, facile electron/mass transport, and desirable OH^−^ adsorption capability. Remarkably, the superiority of phosphorization and the hollow nanocage structure, as well as the synergistic effect between Co and Ni that boosted the electrochemical energy storage performance were demonstrated by controlled experiments and theoretical calculations based on density functional theory (DFT). This work not only provides a promising electrode material for fast energy storage, but also opens a new route toward structural and compositional design of electrode materials for energy storage and conversion.

## 2 Results and Discussion

The synthetic process of NiCo‐P was illustrated in Scheme 1. ZIF‐67 (a Co‐based MOF) nanocrystals with rhombic dodecahedron shape were first synthesized by the coprecipitation reaction of Co^2+^ and 2‐methylimidazole. Scanning electron microscopy (SEM) images, transmission electron microscopy (TEM) images, X‐ray diffraction (XRD) data, and N_2_ sorption measurements confirmed the successful synthesis of ZIF‐67 nanocrystals with particle size of ≈800 nm (Figure S1, Supporting Information). The as‐prepared ZIF‐67 nanocrystals were then used as sacrificial templates to synthesize NiCo double‐layered hydroxides (NiCo‐LDH) by a chemical etching process. During this process, protons generated from the hydrolysis of Ni^2+^ gradually etched the ZIF‐67 crystals and then the released Co^2+^/Co^3+^ coprecipitated with Ni^2+^ to form NiCo‐LDH (Figure S2, Supporting Information).^23^, ^24^, ^25^ SEM and TEM images showed that NiCo‐LDH had hollow nanocage structure with rhombic dodecahedron shape, which was composed of interconnected LDH nanosheets (Figure S2A–D, Supporting Information).

**Figure 1:**
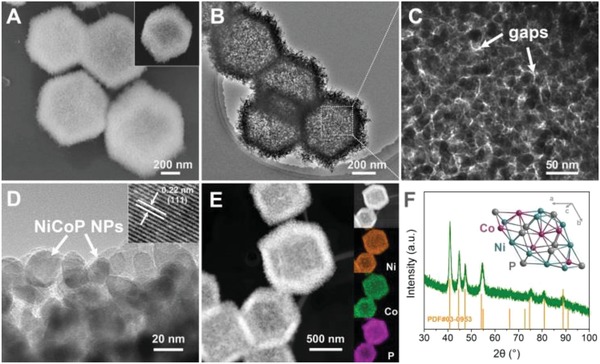
A) SEM image of NiCo‐P. Inset showed a single NiCo‐P nanocage with distinct rhombic dodecahedron shape. B–D) TEM images of NiCo‐P. Inset in (D) showed lattice fringes with spacing of 0.22 nm corresponding to the (111) planes of NiCo‐P. E) HAADF image and the corresponding elemental mapping of NiCo‐P. F) XRD pattern and a structural model of NiCo‐P. The standard XRD pattern of Ni_2_P (PDF#03‐0953) is also shown as reference.

The NiCo‐LDH nanocages were then converted into NiCo‐P after phosphorization using NaH_2_PO_2_ as phosphorus source. During the phosphorization process, PH_3_ generated by decomposition of NaH_2_PO_2_ reacted with NiCo‐LDH to form NiCo‐P.^26^, ^27^ SEM and TEM images of NiCo‐P showed that the rhombic dodecahedron shape and hollow structure were well‐retained after phosphorization (**Figure** 1A). TEM images with higher magnification showed that the NiCo‐P nanocages were composed of interconnected NiCo‐P nanoparticles (Figure 1C,D). The assembly of the interconnected NiCo‐P nanoparticles resulted in abundant gaps (Figure 1C). Distinct lattice fringes with spacing of 0.22 nm can be observed from high‐resolution TEM image, which was corresponding to the (111) planes of NiCo‐P (Figure 1D inset).^28^, ^29^ High‐angle annular dark‐field scanning TEM (HAADF‐STEM) image and the corresponding energy‐dispersive X‐ray spectroscopy (EDS) profiles showed that the Ni, Co, and P were uniformly distributed throughout the whole NiCo‐P nanocages. XRD pattern of NiCo‐P showed characteristic peaks for hexagonal phosphide phase (Ni_2_P type, PDF#03‐0953). It revealed that in NiCo‐P, the Co sites uniformly replaced the Ni sites of Ni_2_P, which was corresponding to previously reported works on NiCo‐P.^30^, ^31^, ^32^

**Figure 2:**
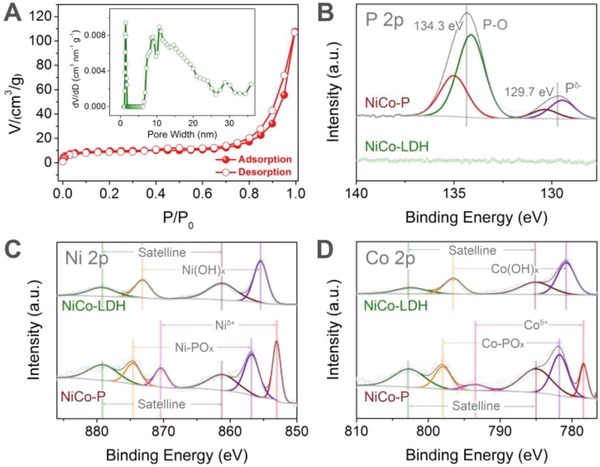
A) N_2_ adsorption/desorption isotherms of NiCo‐P. Inset shows the corresponding pore size distribution. B) High‐resolution P 2p, C) Ni 2p, and D) Co 2p spectra of NiCo‐P and NiCo‐LDH.

N_2_ adsorption/desorption measurements were carried out to investigate the pore structure of NiCo‐P (**Figure** 2A). The N_2_ adsorption/desorption isotherms of NiCo‐P showed distinct hysteresis loop between adsorption and desorption isotherms, and indicated the existence of mesopores (2–50 nm, IUPAC definition).^33^ In addition, the rapidly increasing N_2_ adsorption volume at high relative pressure (*P*/*P*_0_ > 0.9) revealed the presence of macropores (>50 nm), which were originated from the stacking of NiCo‐P nanoparticles. The pore size distribution further revealed the hierarchical porous structure of NiCo‐P, with micropores of ≈1.4 nm and mesopores of various sizes (Figure 2A inset). The hierarchical porous structure may benefit the electrochemical energy storage of NiCo‐P, as the abundant meso/macropores can facilitate mass transport/ion diffusion within the structure while the micropores can provide sufficient spaces and active sites for ion adsorption/electrochemical reactions.^34^

The surface chemical composition of NiCo‐P was investigated by X‐ray photoelectron spectroscopy (XPS) measurements. High‐resolution P 2p XPS spectrum showed that two main peaks at 134.3 and 129.7 eV, which can be assigned to oxidized phosphorus species and metal phosphide, respectively, were observed for NiCo‐P after phosphorization of NiCo‐LDH (Figure 2B). High‐resolution Ni 2p spectrum of NiCo‐P can be divided into component peaks at 870.4 and 853.0 eV corresponding to Ni 2p_1/2_ and 2p_3/2_ of metal phosphide, respectively, as well as peaks at 874.6 and 856.8 eV with two shakeup satellite peaks assigned to oxidized Ni species due to the surface oxidation (Figure 2C).^35^ For comparison, high‐resolution Ni 2p spectrum of NiCo‐LDH only showed peaks for Ni(OH)*_x_*. Similar to the Ni 2p, the high‐resolution Co 2p spectrum of NiCo‐P showed component peaks for metal phosphide (793.5 and 778.4 eV) and oxidized Co species (798.0, 784.9 eV, and satellite peaks, Figure 2D).^30^ The aforementioned XPS results indicated the successful phosphorization of NiCo‐LDH to form NiCo‐P.

**Figure 3:**
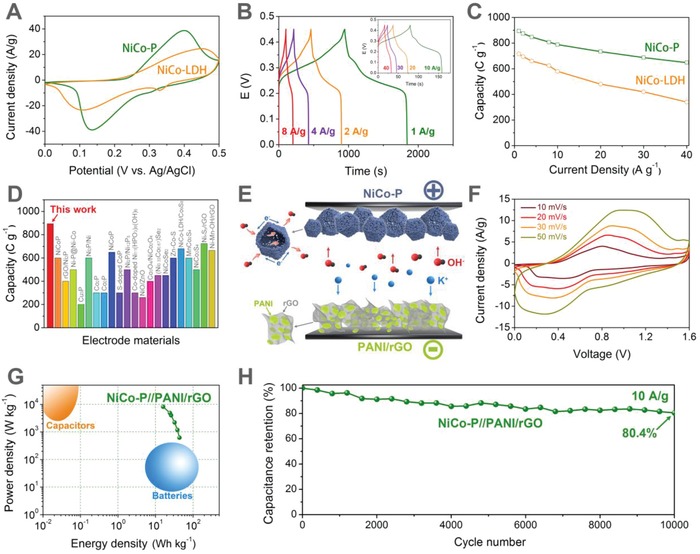
A) CV curves of NiCo‐LDH and NiCo‐P at 10 mV s^−1^. B) GCD curves of NiCo‐P at different current densities. C) Specific capacities of NiCo‐LDH and NiCo‐P at different current densities. D) Comparison of the specific capacities of NiCo‐P and recently reported metal compounds. Detailed information is listed in Table S1 in the Supporting Information. E) Schematic illustration of the hybrid device with NiCo‐P and PANI/rGO as positive and negative electrodes, respectively. F) CV curves of the NiCo‐P//PANI/rGO hybrid device. G) Ragone plot of NiCo‐P//PANI/rGO. Typical domains for capacitors and batteries are shown as references. H) Cycling stability of NiCo‐P//PANI/rGO at a current density of 10 A g^−1^ between 0 and 1.6 V.

The electrochemical performance of NiCo‐P was first evaluated using a three‐electrode configuration in 2 m KOH solution. The cyclic voltammetry (CV) curve of NiCo‐P showed a pair of well‐defined redox peaks at 0.13 and 0.39 V, which can be assigned to Ni*^δ^* ^+^/Ni^3+^ and Co*^δ^* ^+^/Co^4+^ Faradaic reaction (**Figure** 3A).^31^ Moreover, the CV curve of NiCo‐P showed higher current density and larger integral area than those of NiCo‐LDH, indicating that NiCo‐P had a higher specific capacity and facilitated redox reaction kinetics than NiCo‐LDH. The galvanostatic charge‐discharge (GCD) measurements showed that NiCo‐P had nonlinear GCD profiles with potential plateaus between 0.2 and 0.3 V, which was in accordance with the electrochemical behavior of CV measurements (Figure 3B). The CV and GCD measurements indicated that NiCo‐P is a battery‐type electrode material, which is characterized by the well‐separated redox peaks (0.13 and 0.39 V) in CV curve and the distinct voltage plateau (0.2–0.3 V) in the GCD profile, and we used specific capacity in C g^−1^ to describe the electrochemical performance of the battery‐type NiCo‐P material as suggested.^36^, ^37^ The specific capacities calculated from the GCD curves showed that NiCo‐P had a high specific capacity of 894 C g^−1^ at 1 A g^−1^ and a specific capacity of 648 C g^−1^ at high current density of 40 A g^−1^ with a capacity retention of 72.4% (Figure 3C). The outstanding performance of NiCo‐P surpasses most of the reported phosphides and other metal compounds (Figure 3D; Table S1, Supporting Information).In contrast, NiCo‐LDH showed a lower capacity of 716 C g^−1^ at 1 A g^−1^, and less than 50% of the capacity can be retained at 40 A g^−1^. The significantly improved electrochemical performance of NiCo‐P indicated the superiority of phosphorization of NiCo‐LDH to form NiCo‐P as a battery‐type Faradaic electrode material for fast energy storage.

To investigate the practical potential of NiCo‐P, a hybrid supercapacitor device was constructed using NiCo‐P as positive electrode and PANI/rGO as negative electrode (Figure 3E). The electrochemical measurements of the PANI/rGO negative electrode showed both electrical double‐layer and pseudocapacitive behavior between −1 and 0 V (Figure S3, Supporting Information). The charge balance of the positive and negative materials in the hybrid supercapacitor was adjusted by the mass ratio of the materials (Figure S4, Supporting Information). Voltage window of 0–1.6 V was applied for the electrochemical measurements of the NiCo‐P//PANI/rGO hybrid supercapacitor (Figure S5, Supporting Information). The CV curves of the NiCo‐P//PANI/rGO hybrid supercapacitor showed distinct redox peaks and the shapes remained almost the same at elevated scan rates, indicating desirable rate capability (Figure 3F). The GCD curves of the NiCo‐P//PANI/rGO hybrid supercapacitor showed battery‐type sloping voltage plateau and the symmetric shapes reveal high Coulombic efficiencies (Figure S6, Supporting Information). Based on the GCD measurements, Ragone plot that revealed the relationship between power and energy densities is shown in Figure 3G. It showed that compared with conventional capacitors (high power density but low energy density) and rechargeable batteries (high energy density but low power density), the as‐prepared hybrid supercapacitor showed both high energy density and power density. Specifically, the NiCo‐P//PANI/rGO hybrid supercapacitor can deliver an energy density of 43.4 Wh kg^−1^ at power density of 620 W kg^−1^, and an energy density of 16 Wh kg^−1^ at high power density of 8236 W kg^−1^, indicating the excellent performance of the NiCo‐P//PANI/rGO hybrid supercapacitor for fast energy storage. Furthermore, we successfully powered a commercial light‐emitting diode (LED) using two NiCo‐P//PANI/rGO devices connected in series to achieve a voltage window of 3 V, which were capable of powering the commercial LED after being fully charged (Figure S7, Supporting Information). Moreover, it also exhibited excellent cycling stability with capacitance retention of more than 80% after 10 000 cycles at high current density of 10 A g^−1^ (Figure 3H). These results indicated that NiCo‐P can serve as a promising battery‐type Faradaic electrode material to construct hybrid supercapacitor devices for practical fast energy storage applications.

**Figure 4:**
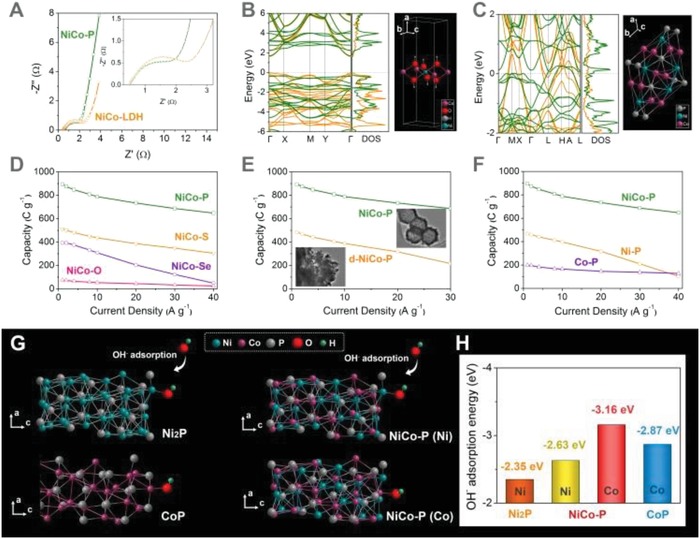
A) Nyquist plots of NiCo‐LDH and NiCo‐P. B,C) Band structures and the corresponding total density of states of (B) NiCo‐LDH and (C) NiCo‐P. The green and orange lines correspond to spin‐up and spin‐down, respectively. D) Specific capacities of NiCo‐P, NiCo‐S, NiCo‐Se, and NiCo‐O at different current densities. E) Specific capacities of NiCo‐P and d‐NiCo‐P at different current densities. F) Specific capacities of NiCo‐P, Ni‐P, and Co‐P at different current densities. G) Schematic illustration of adsorption of OH^−^ on the surface of Ni_2_P, CoP, and NiCo‐P, and H) the corresponding OH^−^ adsorption energy.

The outstanding energy storage performance of NiCo‐P and the superiority of the phosphorization of the MOF‐derived NiCo‐LDH to form NiCo‐P can be attributed to the following reasons: 1) The phosphorization of NiCo‐LDH to form NiCo‐P can lead to enhanced electrical conductivity, which was evidenced by electrochemical impedance spectroscopy measurements (**Figure** 4A). The Nyquist plots of NiCo‐P showed a smaller diameter of the semicircle at high frequency region and a straight line closer to the imaginary axis compared with NiCo‐LDH, indicating much facilitated charge transfer kinetics after the phosphorization transformation of NiCo‐LDH to form NiCo‐P.^12^ Moreover, DFT calculations were carried out to study the electronic band structures of NiCo‐P and NiCo‐LDH (Figure 4B,C). It showed that NiCo‐P was metallic, while NiCo‐LDH was semiconductor with a band gap of 1.9 eV, further indicating the better electrical conductivity of NiCo‐P than NiCo‐LDH. 2) The unique chemical composition of the NiCo‐P with metal‐rich Ni_2_P structure can provide abundant redox active sites.^22^ To demonstrate the superiority of the phosphorization process, NiCo‐LDH was transformed into NiCo‐S, NiCo‐Se, and NiCo‐O by sulfidation, selenation, and oxidation of the as‐prepared NiCo‐LDH, respectively (Figures S8–S10, Supporting Information). It was found that these control samples showed much poorer electrochemical performance compared with NiCo‐P, with much lower specific capacities at 1 A g^−1^ and greater capacity losses at high current densities, which indicated the superiority of phosphorization of NiCo‐LDH for fast energy storage (Figure 4D; Table S2, Supporting Information). 3) The utilization of the MOF crystals of ≈800 nm as precursors led to the formation of well‐defined hollow nanocage morphology composed of interconnected NiCo‐P nanoparticles with abundant gaps, which can facilitate ion transport/diffusion. For comparison, disordered NiCo‐P (d‐NiCo‐P) without well‐defined nanocage morphology was synthesized following the same synthetic procedure except that MOF crystals with much smaller size (≈100 nm) were used as precursors (Figures S11–S14, Supporting Information). It was found that d‐NiCo‐P showed much lower capacity (484 C g^−1^ at 1 A g^−1^) and poorer rate capability (with capacity retention of less than 45% at 30 A g^−1^), which revealed that rational morphology design of the NiCo‐P had a pivotal role in fast energy storage applications (Figure 4E). Although the hollow porous structure of NiCo‐P can provide many advantages including facilitated mass transport and high exposure of electrochemical active sites, it should be noted that the large void may also lead to low packing density and low volumetric energy density. Further optimization of the pore structure and the morphology may achieve a balance between gravimetric and volumetric energy density.^38^ 4) The uniformly incorporated Co sites can greatly enhance the electrochemical performance of the NiCo‐P due to the synergistic effect between Co and Ni. By adjusting the experimental conditions during the formation of LDH, Co‐P and Ni‐P were synthesized, which showed inferior electrochemical energy storage performances compared with NiCo‐P (Figure 4F). The superior performance of NiCo‐P was attributed to the synergistic effect between Co and Ni. DFT calculations were carried out to demonstrate the role of the incorporated Co sites (Figure 4G,H). It was proposed that the OH^−^ adsorption energy can reveal the redox reaction charge–discharge kinetics, which will greatly affect the ion transfer efficiency between active materials and electrolyte, therefore the OH^−^ adsorption energies on the surface of Ni_2_P, CoP, and NiCo‐P was calculated (Figure 4G,H).^12^ It was found that the OH^−^ adsorption energy of the Ni sites of NiCo‐P (−2.63 eV) was lower than that of Ni‐P (−2.35 eV), indicating more facile OH^−^ adsorption at the Ni sites of NiCo‐P in KOH electrolyte. Moreover, the incorporated Co sites themselves can serve as OH^−^ adsorption sites and have an OH^−^ adsorption energy of −3.16 eV, lower than that of the CoP (−2.87 eV), revealing that the synergistic effect between Ni and Co can improve the OH^−^ adsorption capability of both Ni and Co. The improved OH^−^ adsorption capability may be attributed to the modification of the electronic structure of the Ni/Co sites, which was evidenced by the reduced electron density around the Ni/Co sites (Figure S15, Supporting Information). These abovementioned results showed that in addition to structural design, the rational design of the chemical composition of NiCo‐P also contributed to its outstanding energy storage performance.

## 3 Conclusion

In conclusion, we presented an MOF‐based strategy to fabricate NiCo‐P hollow nanocage as a battery‐type Faradaic electrode material for fast energy storage. The outstanding fast energy storage performance of NiCo‐P was attributed to its advantageous structural and chemical compositional design that led to improved electrical conductivity, facile electron/mass transport, and desirable OH^−^ adsorption capability, which was demonstrated by control experiments and theoretical calculations based on DFT that revealed the synergistic effect between Ni and Co in NiCo‐P can greatly increase the OH^−^ adsorption energy, while the hollow porous structure facilitated the fast mass/electron transport. We believe this work not only provides a new route toward structural and compositional design of electrode materials for energy storage and conversion, but also offers guidance and inspiration to future works on various energy storage and conversion applications.

## Conflict of Interest

The authors declare no conflict of interest.

## Supporting information

Supplementary
