# Supplementary material for: Synergistic Effect of Co–Ni Hybrid Phosphide Nanocages for Ultrahigh Capacity Fast Energy Storage
Source: Adv Sci (Weinh). 2019 Feb 20;6(8):1802005. doi: 10.1002/advs.201802005 (PMC6469242; doi:10.1002/advs.201802005)
Supplement: Supplementary file 1 — Supplementary [file ADVS-6-1802005-s001.pdf]

Copyright WILEY-VCH Verlag GmbH & Co. KGaA, 69469 Weinheim, Germany, 2019.

ADVANCED SCIENCE
Open Access

Supporting Information

for *Adv. Sci.,* DOI: 10.1002/advs.201802005

Synergistic Effect of Co–Ni Hybrid Phosphide Nanocages for Ultrahigh Capacity Fast Energy Storage

Zibin Liang, Chong Qu, Wenyang Zhou, Ruo Zhao, Hao Zhang, Bingjun Zhu, Wenhan Guo, Wei Meng, Yingxiao Wu, Waseem Aftab, Qian Wang, and Ruqiang Zou**

Copyright WILEY-VCH Verlag GmbH & Co. KGaA, 69469 Weinheim, Germany, 2019.

Supporting Information

Synergistic effect of Co-Ni hybrid phosphide nanocages for ultrahigh capacity fast energy storage

Zibin Liang, Chong Qu, Wenyang Zhou, Ruo Zhao, Hao Zhang, Bingjun Zhu, Wenhan Guo, Wei Meng, Yingxiao Wu, Waseem Aftab, Qian Wang,* and Ruqiang Zou*

Chemicals and materials

$Co(NO_3)_2 \cdot 6H_2O$ was purchased from Xilong Scientific Co., Ltd.. $Ni(NO_3)_2 \cdot 6H_2O$ and polyvinylpyrrolidone (PVP) were purchased from Sinopharm Chemical Reagent Co., Ltd.. 2-methylimidazole was purchased from Beijing Ouhe Technology Co., Ltd.. NaH_2PO_2 was obtained from Shanghai Macklin Biochemical Co., Ltd.. All the reagents were directly used without further treatment.

Experimental Methods

Synthesis of ZIF-67 nanocrystals with size of ~800 nm: The ZIF-67 nanocrystals with size of ~800 nm were synthesized using a co-precipitation method. Typically, 1.455 g of $Co(NO_3)_2 \cdot 6H_2O$ was dissolved into 100 mL of methanol to form solution A. 1.64 g of 2-methylimidazole was dissolved into 100 mL of methanol to form solution B. Afterwards, solution B was poured into solution A under vigorous stirring. The resultant mixture was kept stirring for 10 min, and was then kept standing for 24 h.

The resultant ZIF-67 nanocrystals were separated by centrifugation and washed with ethanol for 3 times, followed by drying at 80 °C for 12 h.

Synthesis of ZIF-67 nanocrystals with size of ~100 nm: ZIF-67 nanocrystals with size of ~100 nm were synthesized for comparison. Typically, 1.176 g of $Co(NO_3)_2·6H_2O$ and 1 g of polyvinylpyrrolidone (PVP) were dissolved into 100 mL of methanol to form solution A. 1.325 g of 2-methylimidazole and 0.08 mL of triethylamine were dissolved into 100 mL of methanol to form solution B. Solution B was then poured into solution A under vigorous stirring. After standing for 24 h, the resultant ZIF-67 nanocrystals were separated by centrifugation and washed with ethanol for 3 times, followed by drying at 80 °C for 12 h.

Synthesis of NiCo-LDH: Typically, 50 mg of the as-prepared ZIF-67 nanocrystals with size of ~800 nm were dispersed in 10 mL of ethanol by sonication. Then 5 mL of ethanol containing 200 mg of $Ni(NO_3)_2·6H_2O$ was injected into the ZIF-67 suspension. The resultant mixture was transferred in a 20 mL Teflon-lined autoclave and was heated at 120 °C for 4 h. NiCo-LDH was then separated by centrifugation and washed with ethanol for 3 times, followed by drying at 80 °C for 12 h.

Synthesis of d-NiCo-LDH: For comparison, disordered NiCo-LDH (d-NiCo-LDH) without well-defined nanocage hollow structure was synthesized using ZIF-67 nanocrystals with size of ~100 nm as precursors. Typically, 50 mg of the as-prepared

ZIF-67 nanocrystals with size of ~100 nm were dispersed in 10 mL of ethanol by sonication. Then 5 mL of ethanol containing 200 mg of $Ni(NO_3)_2 \cdot 6H_2O$ was injected into the ZIF-67 suspension. The resultant mixture was transferred in a 20 mL Teflon-lined autoclave and was heated at 120 °C for 4 h. d-NiCo-LDH was then separated by centrifugation and washed with ethanol for 3 times, followed by drying at 80 °C for 12 h.

Synthesis of NiCo-P and d-NiCo-P: Typically, 10 mg of the as-prepared NiCo-LDH was placed in a small crucible, and 200 mg of NaH_2PO_2 was placed in a bigger crucible. The small crucible containing NiCo-LDH was place in the bigger one containing NaH_2PO_2 which was then transferred into a tube furnace and heated at 350 °C for 2 h under Ar atmosphere with a temperature ramping rate of 2 °C/min, and NiCo-P was obtained. Disordered NiCo-P (d-NiCo-P) without well-defined nanocage hollow structure was obtained following the same method, except that d-NiCo-LDH was used as precursor.

Synthesis of NiCo-S, NiCo-O, and NiCo-Se: For comparison, NiCo-S, NiCo-O, and NiCo-Se were synthesized by sulfidation, oxidation, and selenization of the as-prepared NiCo-LDH, respectively. For sulfidation of NiCo-LDH, NiCo-LDH was treated at 350 °C for 2 h under H_2S/Ar (v:v=1:99) atmosphere with a temperature ramping rate of 2 °C/min. For oxidation of NiCo-LDH, NiCo-LDH was treated at 400 °C for 2 h in air with a temperature ramping rate of 1 °C/min. For selenization of

NiCo-LDH, 10 mg of the as-prepared NiCo-LDH was placed in a small crucible, and 500 mg of Se powder was placed in a bigger crucible. The small crucible containing NiCo-LDH was placed in the bigger one containing Se powder, which was then transferred into a tube furnace and heated at 500 °C for 2 h under Ar atmosphere with a temperature ramping rate of 2 °C/min.

Synthesis of Co-P and Ni-P: In order to minimize the Ni or Co concentration in the resultant Co-LDH or Ni-LDH, ZIF-67 nanocrystals were treated with $Co(NO_3)_2{\cdot}6H_2O$ or excess $Ni(NO_3)_2{\cdot}6H_2O$. Typically, 50 mg of the as-prepared ZIF-67 nanocrystals with size of ~800 nm were dispersed in 10 mL of ethanol by sonication. Then 5 mL of ethanol containing 200 mg of $Co(NO_3)_2{\cdot}6H_2O$ (for Co-LDH) or 75 mL of ethanol containing 4 g of $Ni(NO_3)_2{\cdot}6H_2O$ (for Ni-LDH) was injected into the ZIF-67 suspension. The resultant mixture was transferred in a Teflon-lined autoclave and was heated at 120 °C for 4 h. Co-LDH or Ni-LDH was then separated by centrifugation and washed with ethanol for 3 times, followed by drying at 80 °C for 12 h. Co-P and Ni-P were obtained following the same method used for synthesis of NiCo-P, except that Co-LDH and Ni-LDH were used as precursor, respectively.

Characterization: XRD data was measured by Rigaku Corporation SmartLab 9 kW at 45 kV and 200 mA using Cu Kα radiation. SEM images were measured on a Hitachi S-4800 microscope. TEM images were measured using a transmission electron microscope (Hitachi, H-9000NAR). HRTEM were measured using a FEI Tecnai F30

microscope equipped with an energy-dispersive X-ray (EDX) detector. XPS was measured by X-ray photoelectron spectroscopy (XPS, Axis Ultra, Kratos Analytical, Japan) with monochromatic aluminum Kα as source of X-ray. The nitrogen adsorption/desorption isotherms of the materials were measured within the pressure range 0-1 atm at 77K using a Quadrasorb system from a Quantachrome Autosorb-IQ gas adsorption analyzer. Non-local density functional theory (NL-DFT) method was used to measure the pore size distribution.

Electrochemical measurements: Except that EIS measurements were conducted in a Zahner Zennium electrochemical workstation, all the other electrochemical tests were carried out in a CHI 760E electrochemical workstation. For three-electrode configuration tests, an Ag/AgCl (saturated KCl) electrode was used as reference electrode, a Pt foil electrode was used as counter electrode, and 2 M KOH was used as electrolyte. The working electrode was prepared using the following method: a mixture slurry containing of 80 wt% active material, 10 wt% Super P, and 10 wt% PTFE binder was made, which was then rolled with the assistance of ethanol to make a piece of uniform film with a typical areal mass of ~2.5 mg cm^{-2}. The film was dried at 60 ˚C for 12 h under vacuum, and was then pressed between two nickel foam current collectors to obtain the working electrode. The cyclic voltammetry (CV) curves were tested between 0 and 0.5 V at different scan rates, and the galvanostatic charge-discharge (GCD) measurements were carried out between 0 and 0.45 V at different current densities in 2 M KOH aqueous electrolyte. Based on the

galvanostatic discharge curve, the specific capacities Q (C/g) of the battery-type Faradaic electrode materials were calculated using the following equation:

Q $= i_m \Delta t$

where i_m=I/m (A/g) is the current density, m is the mass of the active material, Δt (s) is the discharge time.

For two-electrode configuration tests, NiCo-P and PANI/rGO were used as positive and negative materials, respectively. The mass ratio of negative to positive materials (m_-:m_+) was calculated based on charge balance theory ($q^+ = q^-$). Based on the CV analysis,

$q = \int imdV/v$

where q is the charge, m represents the mass of the active material, and $\int idV/v$ is the integral area of the CV curves.

To achieve charge balance, $m^+ \cdot \left(\int \frac{idV}{v}\right)_+ = m^- \cdot \left(\int \frac{idV}{v}\right)_-$, therefore,

$$m^+ : m^- = \left(\int \frac{idV}{v}\right)_- : \left(\int \frac{idV}{v}\right)_+$$

Specifically, the mass ratio of negative to positive materials (m_-:m_+) was calculated to be ~2.6. For the full cell NiCo-P//PANI/rGO, the total weight of NiCo-P (~2 mg/cm^2) and PANI/rGO (~5.2 mg/cm^2) electrodes is ~7.2 mg/cm^2.

The energy density E (Wh/kg) and power density P (W/kg) in Ragone plot were calculated with the following equations,

$$\mathrm{E} = \frac{\mathrm{i_m} \int \mathrm{Vdt}}{3.6}$$

$$\mathrm{P} = 3600 \cdot \frac{\mathrm{E}}{\Delta \mathrm{t}}$$

Where $\int Vdt$ is the integral current area, i_m=I/m is the current density, where I is the current and m is the mass of active materials, and Δt is the discharge time (s).

Theoretical calculation method:

All calculations were performed based on DFT as implemented in Vienna Ab-initio Simulation Package (VASP).[1-2] Electron wave functions were expanded by using the projector augmented wave (PAW) method with a kinetic energy cutoff of 400 eV. The Perdew-Burke-Ernzerhof (PBE) functional[3] for the generalized gradient approximation (GGA) was used to treat the electron exchange-correlation interaction. For bulk NiCo-P and 2D NiCo-LDH, Monkhorst-Pack sampling[4] of 6x6x10 and 10x10x1 were used and all atoms are fully relaxed, We have taken into account the Coulomb correction for Co 3d and Ni 3d electrons by using LSDA+U method with the Hubbard U values of 5.1 and 6.4 for Co and Ni, respectively. which have been used in some similar systems[5] To calculate the adsorption energy of OH^- on Ni_2P, CoP, and NiCoP surfaces, we used supercell approach where Ni_2P, CoP, and NiCoP were modeled by using (2x2) six-layer slab supercells having the (111) surface orientation, respectively. The supercells of both Ni_2P and NiCoP contain 54 atoms while that of CoP contains 48 atoms. The bottom four layers of the slabs were held at their bulk crystalline positions, while the top two layers of the slabs were allowed to relax without any symmetry constraint. Each slab was separated from the other by a vacuum region of 12 Å along the (111) direction. (5x5x1) and (10x10x1) Monkhorst-Pack grid, respectively, for the structure optimization and for the total

energy calculations were used. In all the calculations, self-consistency was achieved by allowing the total energy to converge with 0.0001 eV. The Hellman-Feynman force components on each ion in the slab supercells are converged to 0.01 eV/Å.

The adsorption energy of OH^- was calculated using the formula below:

$$E_{ads} = E_{*-OH} - E_{slab} - E_{OH^-},$$

where E_{*-OH}, E_{slab}, and E_{OH^-} are the total energies of the slab with OH^- adsorbed on the Ni site, the slab supercell, and an isolated OH^-, respectively. Meanwhile, Dipole corrections are also considered in those calculations of adsorption energy.[6]

Physical and electrochemical characterizations

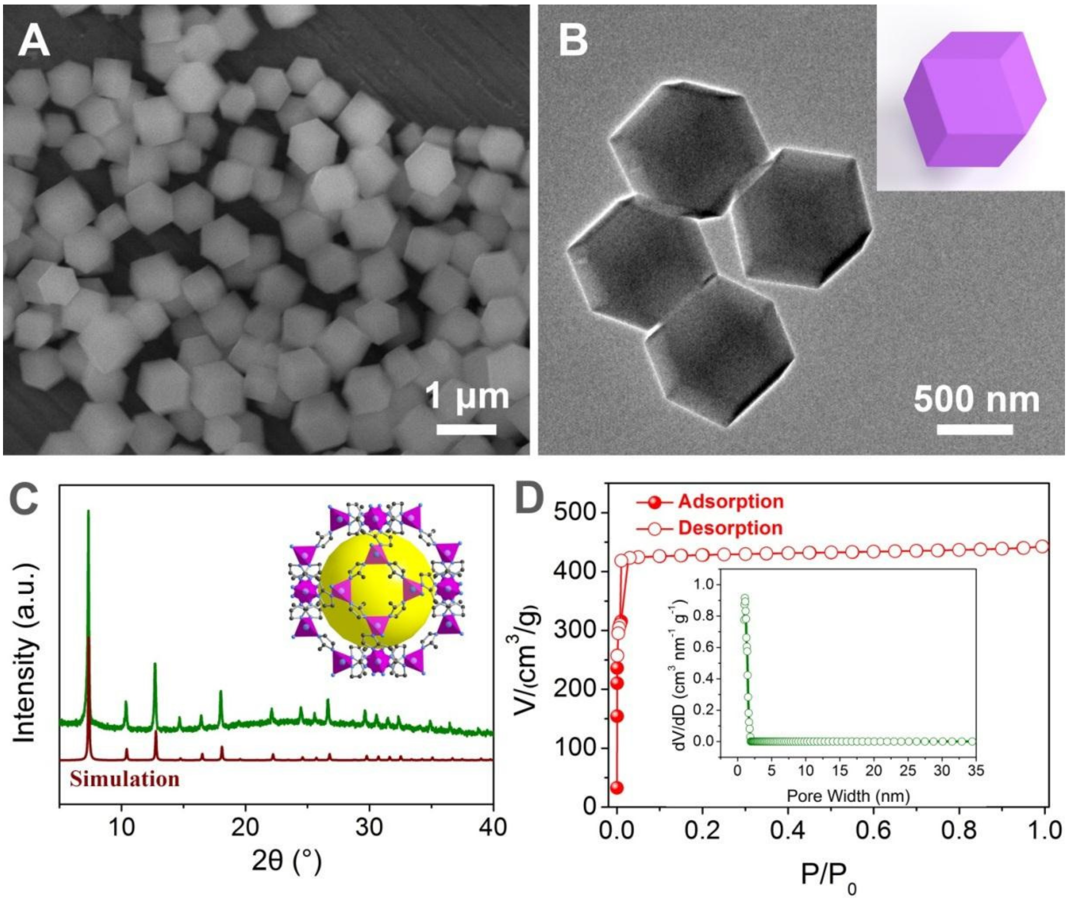

Figure S1. Characterizations of the as-prepared ZIF-67 nanocrystals with size of ~800 nm. (A) SEM and (B) TEM images showed well-defined rhombic dodecahedron morphology of the ZIF-67 nanocrystals. Inset in (B) showed a schematic model of a rhombic dodecahedral ZIF-67 crystal. (C) XRD pattern of the as-prepared ZIF-67. The simulation XRD pattern was also showed as reference. Inset showed a part of the crystal structure of ZIF-67. (D) N_2 adsorption/desorption isotherms and the corresponding pore size distribution of the as-prepared ZIF-67.

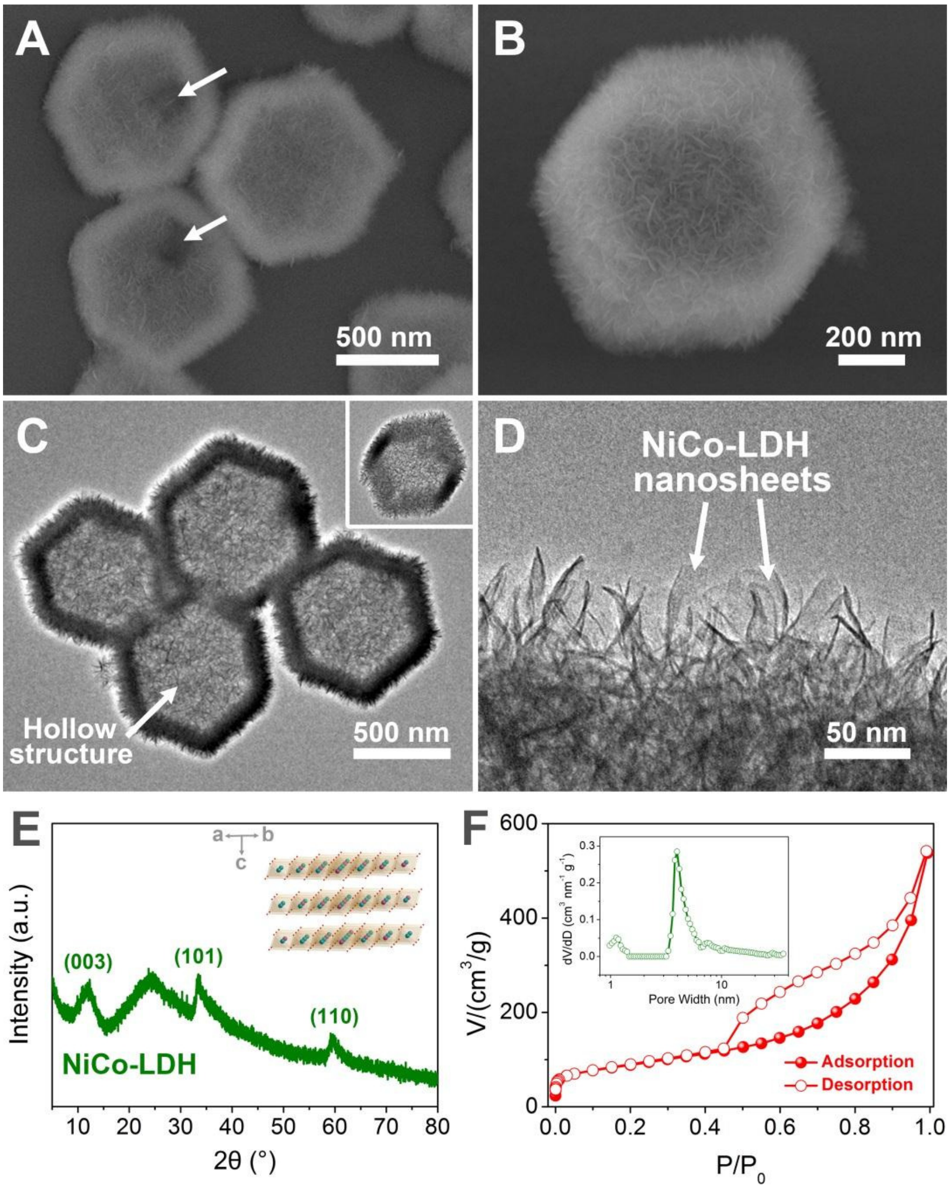

Figure S2. Characterizations of the as-prepared NiCo-LDH nanocages. (A-B) SEM images of NiCo-LDH. Arrows in (A) pointed out the hollow structure of the broken NiCo-LDH nanocages. (C-D) TEM images of NiCo-LDH showing that the hollow NiCo-LDH nanocages were composed of nanosheets. Inset in (C) showed a NiCo-LDH nanocage with distinct rhombic dodecahedron shape. (E) XRD pattern of NiCo-LDH. Inset showed a schematic structural model of NiCo-LDH. (F) N_2 adsorption/desorption isotherms and the corresponding pore size distribution of NiCo-LDH, which revealed that NiCo-LDH had hierarchical porous structure.

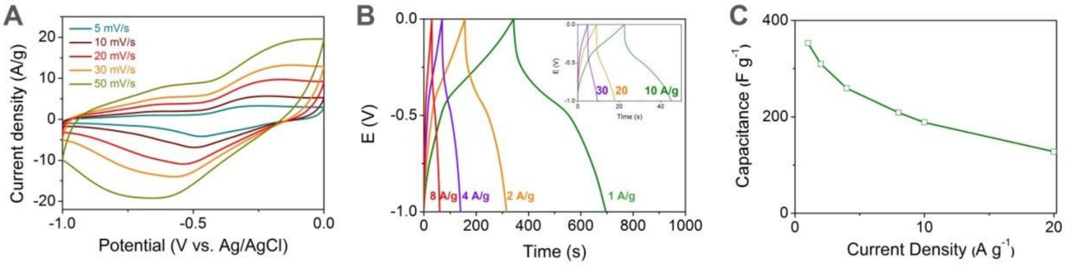

Figure S3. Electrochemical measurements of the as-prepared PANI/rGO negative electrode. (A) CV curves at different scan rates. (B) GCD curves at different current densities. (C) Capacitance at different current densities calculated from (B).

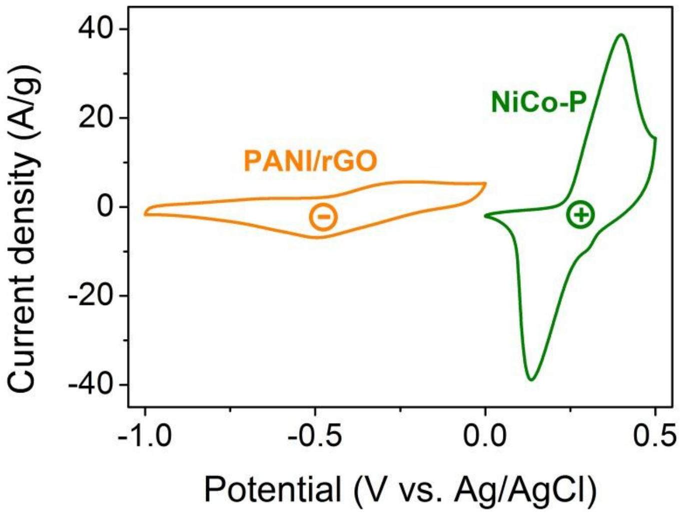

Figure S4. CV comparison of the negative (PANI/rGO) and positive (NiCo-P) electrodes of the hybrid supercapacitor device.

The mass ratio of negative to positive materials (m_-:m_+) was calculated based on charge balance theory ($q^+ = q^-$). Based on the CV analysis,

$q = \int imdV/v$

where q is the charge, m represents the mass of the active material, and $\int idV/v$ is the integral area of the CV curves.

To achieve charge balance, $m^+ \cdot \left(\int \frac{idV}{v}\right)_+ = m^- \cdot \left(\int \frac{idV}{v}\right)_-$, therefore,

$$m^+ : m^- = \left(\int \frac{idV}{v}\right)_- : \left(\int \frac{idV}{v}\right)_+$$

Specifically, the mass ratio of negative to positive materials (m_-:m_+) was calculated to be ~2.6.

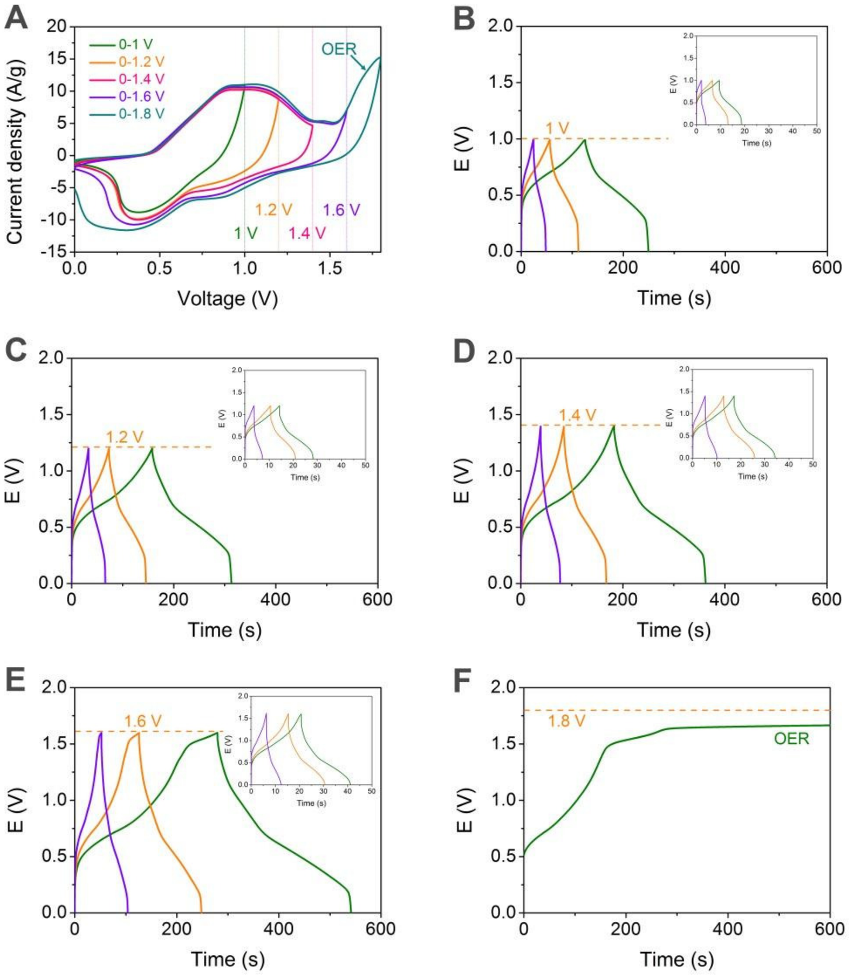

Figure S5. (A) CV curves of NiCo-P//PANI/rGO tested within different voltage windows. (B-E) GCD curves of NiCo-P//PANI/rGO measured within different voltage windows: (B) 0-1 V, (C) 0-1.2 V, (D) 0-1.4 V, and (E) 0-1.6V. (F) Charging curve of NiCo-P//PANI/rGO tested within a voltage window of 0-1.8 V.

For CV curves measured within 0-1 V, 0-1.2 V, and 0-1.4V, the voltage window did not fully cover the potentials of the redox reaction while for an extended voltage window (0-1.8 V), a rapidly increasing current density due to the oxygen evolution reaction (OER) was observed. Therefore, voltage window of 0-1.6 V was selected for the electrochemical measurements of NiCo-P//PANI/rGO. Besides CV measurements,

GCD curves measured within different voltage also revealed that small voltage windows did not fully cover the potentials of the redox reaction as the charge/discharge processes were not complete, which led to a much lower charge/discharge time compared with that tested within a suitable voltage window (0-1.6 V). For the voltage window of 0-1.8 V, a horizontal straight line was observed and the voltage is difficult to charge to 1.8 V due to the OER.

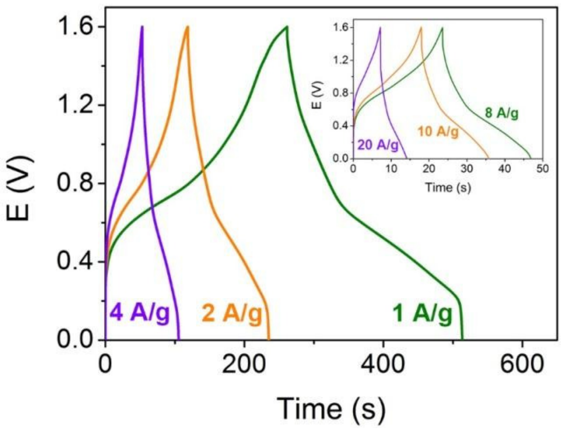

Figure S6. GCD curves of the NiCo-P//PANI/rGO hybrid device at different current densities.

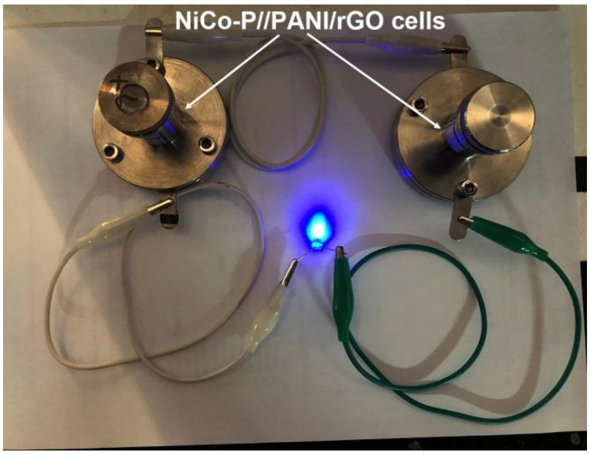

Figure S7. Digital photograph of a commercial light-emitting diode (LED) lighted up by two NiCo-P//PANI/rGO devices connected in series.

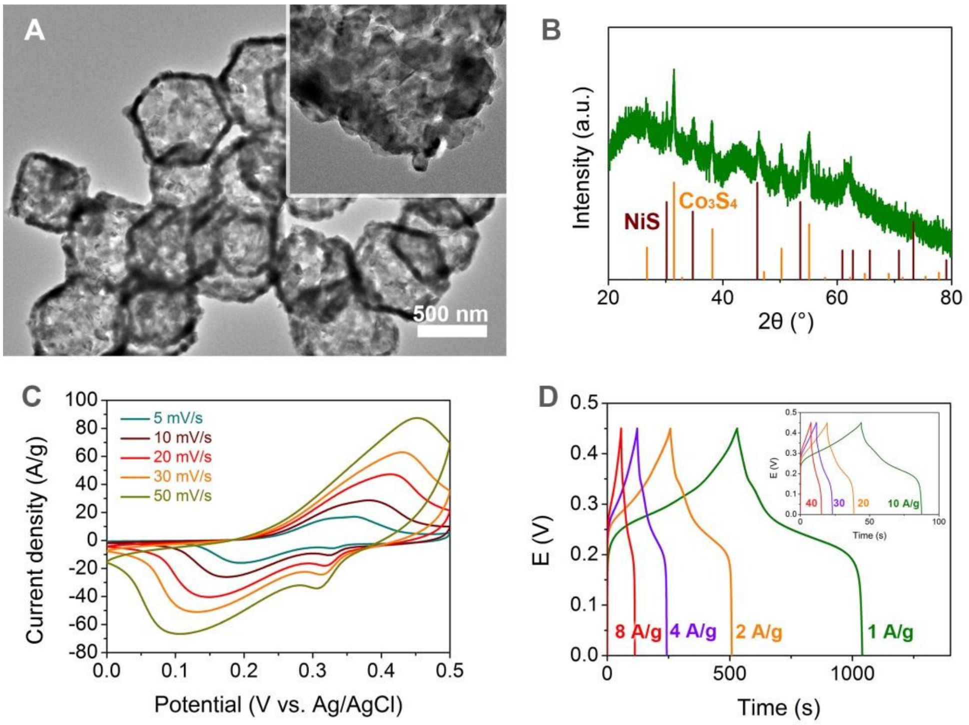

Figure S8. Characterizations of NiCo-S. (A) TEM image. (B) XRD pattern. (C) CV curves at different scan rate. (D) GCD curves at different current densities.

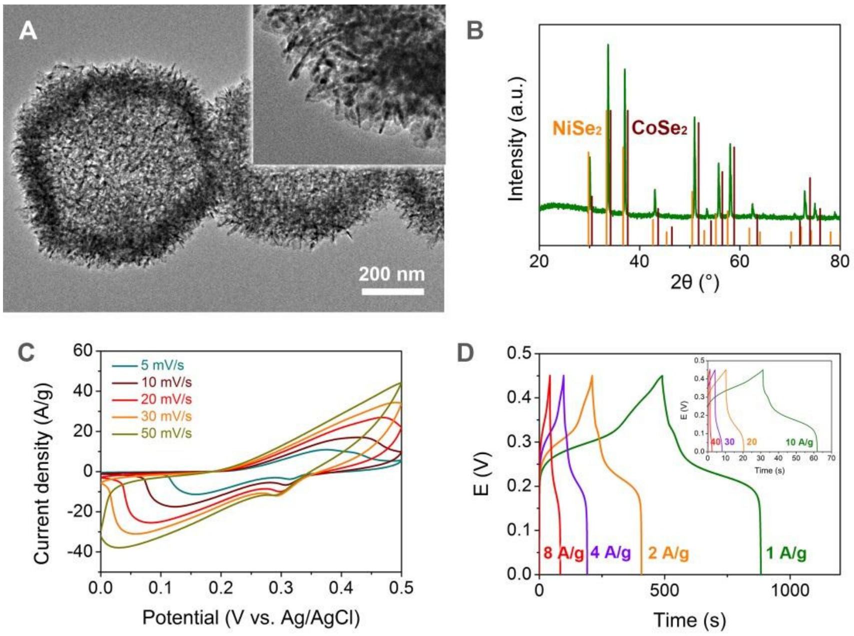

Figure S9. Characterizations of NiCo-Se. (A) TEM image. (B) XRD pattern. (C) CV curves at different scan rate. (D) GCD curves at different current densities.

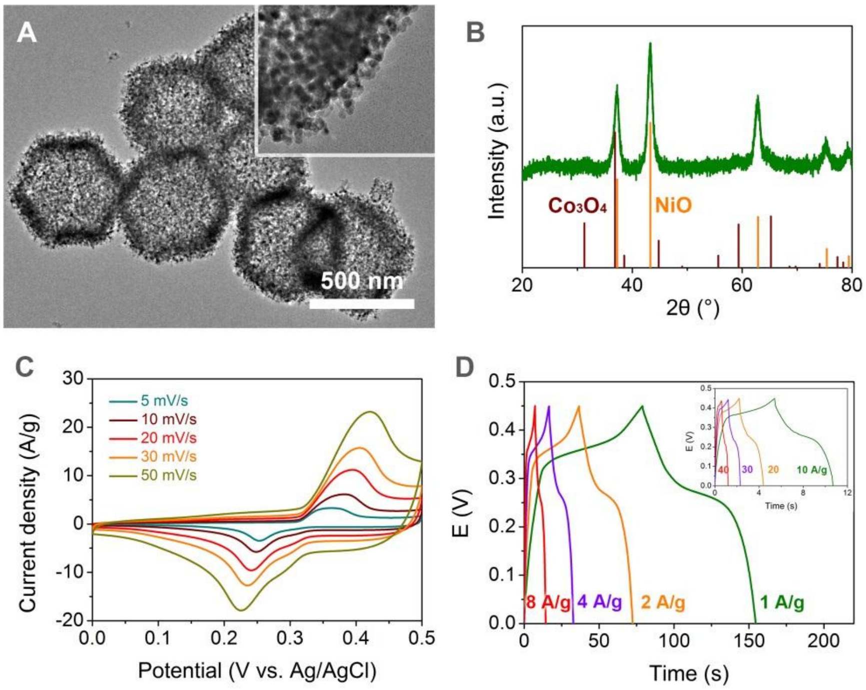

Figure S10. Characterizations of NiCo-O. (A) TEM image. (B) XRD pattern. (C) CV curves at different scan rate. (D) GCD curves at different current densities.

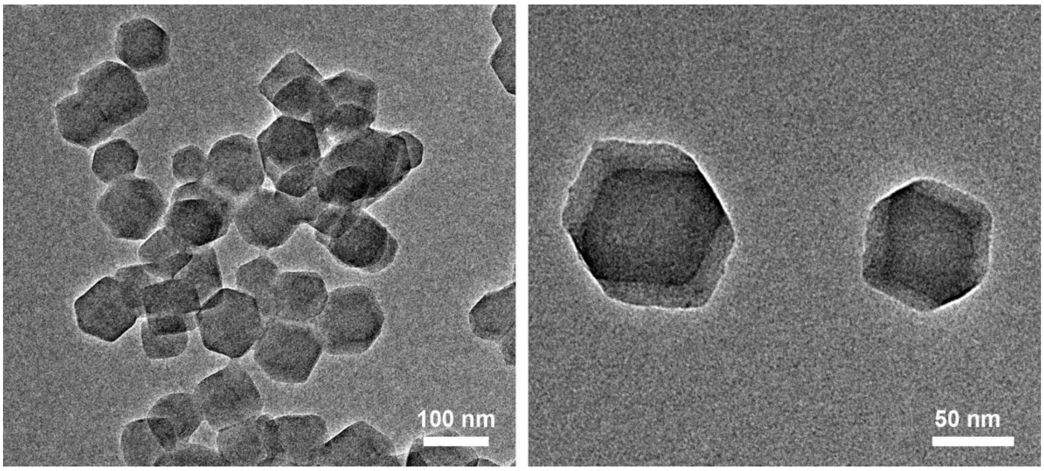

Figure S11. TEM images of ZIF-67 nanocrystals with size of ~100 nm.

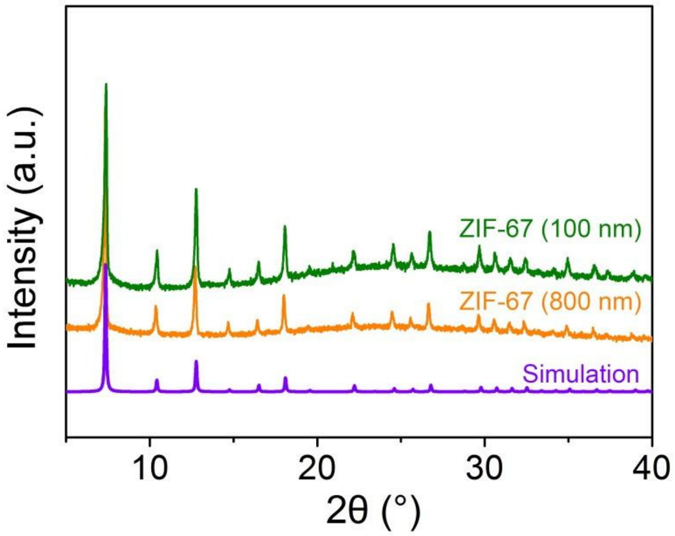

Figure S12. XRD patterns of ZIF-67 nanocrystals with size of ~100 nm. The simulation XRD pattern and the XRD pattern of ZIF-67 nanocrystals with size of ~800 nm were also showed as reference.

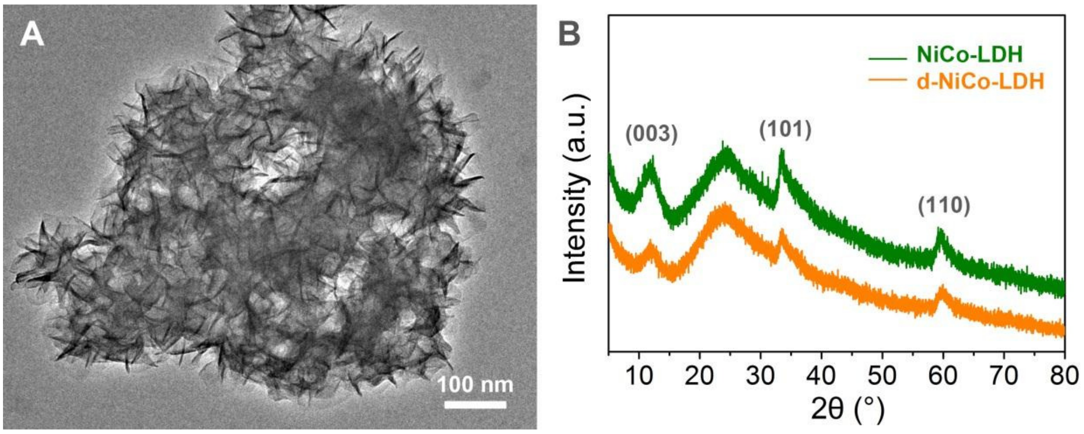

Figure S13. Characterizations of disordered NiCo-LDH (d-NiCo-LDH) derived from ZIF-67 nanocrystals with size of ~100 nm. (A) TEM image showed that hollow nanocage morphology was not formed using ZIF-67 nanocrystals with size of ~100 nm as precursors. (B) XRD pattern of d-NiCo-LDH showed similar characteristic peaks to NiCo-LDH.

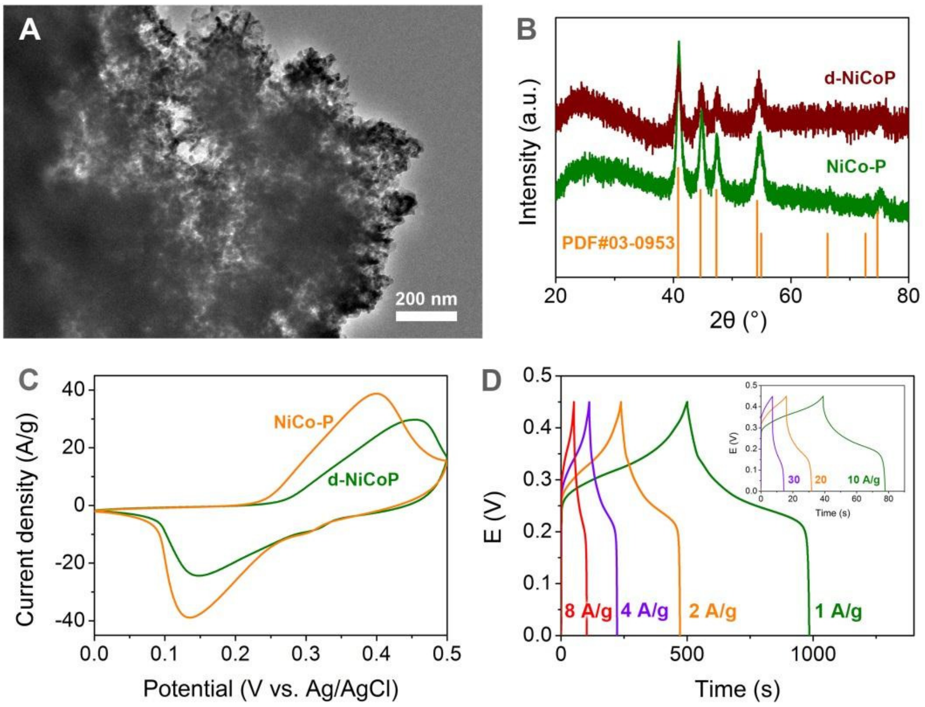

Figure S14. Characterizations of d-NiCo-P. (A) TEM image showed that d-NiCo-P did not have well-defined hollow nanocage structure. (B) XRD pattern of d-NiCo-P showed similar characteristic peaks to NiCo-P. (C) CV curve of d-NiCo-P showed lower current density and smaller integral area than those of NiCo-P. (D) GCD curves at different current densities.

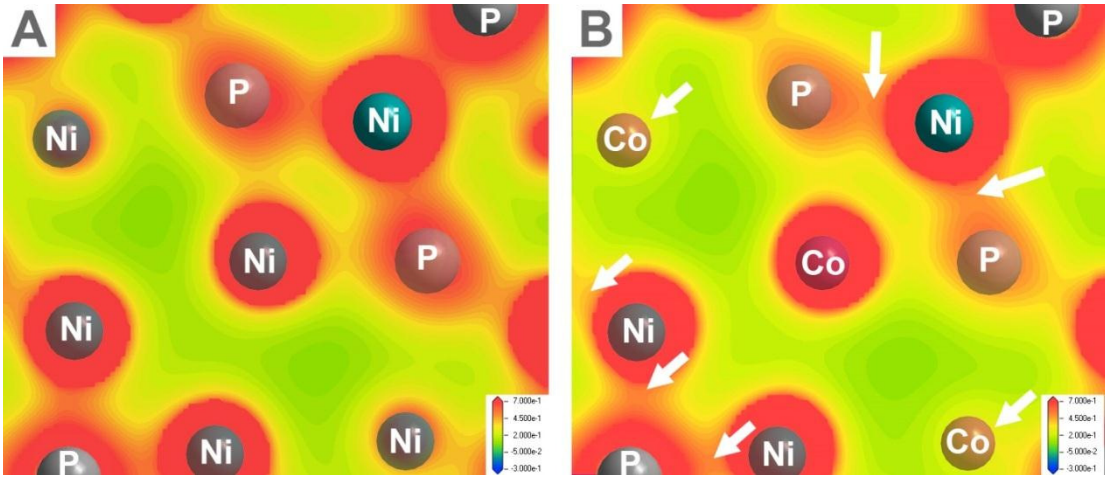

Figure S15. Electron density distribution of Ni_2P (left) and NiCo-P (right). The white arrows point out the reduced electron density around Ni and Co sites compared with Ni_2P.

Table S1. Comparison of the electrochemical performances of NiCo-P and recently reported metal compounds.

Metal compounds	Electrolyte	Rate (A/g)	Capacity (C/g)	Reference
**NiCo-P**	2 M KOH	1	894	This work
**NiCoP**	1 M KOH	1	~600*	[7]
**rGO/Ni_2P**	6 M KOH	2	~400*	[8]
**Ni–P@Ni–Co**	6 M KOH	1	~500*	[9]
**Cu_3P**	2 M KOH	1	~200*	[10]
**Ni_2P/Ni**	2 M KOH	1	~600*	[11]
**Co_2P**	6 M KOH	1	~300*	[12]
**Co_2P**	6 M KOH	1	~300*	[13]
**NiCoP**	2 M KOH	1	~650*	[14]
**S-doped CoP**	6 M KOH	5	~300*	[15]
**$Ni_2P/Ni_{12}P_5$**	2 M KOH	1	~500*	[16]
**Co-doped $Ni_{11}(HPO_3)_8(OH)_6$**	3 M KOH	0.5	~300*	[17]
**NiO/ZnO**	3 M KOH	1.3	~260*	[18]
**$Co_3O_4/NiCo_2O_4$**	2 M KOH	5	~400*	[19]
**$(Ni_{0.33}Co_{0.67})Se_2$**	3 M KOH	1	~450*	[20]
**$NiCoSe_2$**	6 M KOH	3	~450*	[21]
**Zn-Co-S**	6 M KOH	1	~600*	[22]

**NiCo-LDH/Co_9S_8**	1 M KOH	4	~680*	[23]
**$MnCo_2S_4$**	2 M KOH	5	~600*	[24]
**$NiCo_2S_4$**	6 M KOH	2	~500*	[25]
**Ni_xS_y/rGO**	2 M KOH	1	724	[26]
**Ni-Mn-OH/rGO**	2M KOH	2	665	[27]

*Read from the charge-discharge profiles.

Table S2. Comparison of the electrochemical performances of NiCo-P, NiCo-S, NiCo-Se, and NiCo-O.

	**Capacity at 1A/g**	**Retention at 20 A/g**	**Retention at 40 A/g**
**NiCo-P**	894	82%	72%
**NiCo-S**	511	76%	59%
**NiCo-Se**	394	52%	12%
**NiCo-O**	76	58%	32%

[1] G. Kresse, J. Furthmüller, Physical Review B **1996**, 54, 11169-11186.
[2] G. Kresse, D. Joubert, Physical Review B **1999**, 59, 1758.
[3] J. P. Perdew, K. Burke, M. Ernzerhof, Physical review letters **1996**, 77, 3865.
[4] H. J. Monkhorst, J. D. Pack, Physical review B **1976**, 13, 5188.
[5] V. L. Chevrier, S. P. Ong, R. Armiento, M. K. Y. Chan, G. Ceder, Physical Review B **2010**, 82.
[6] L. Bengtsson, Physical Review B **1999**, 59, 12301-12304.
[7] H. Liang, C. Xia, Q. Jiang, A. N. Gandi, U. Schwingenschlögl, H. N. Alshareef, Nano Energy **2017**, 35, 331-340.
[8] Z. Lv, Q. Zhong, Y. Bu, Applied Surface Science **2018**, 439, 413-419.

[9] D. Li, Y. Li, Z. Xu, D. Wang, T. Wang, J. Zhao, H. Zhang, Journal of Materials Science **2018**, 53, 3647-3660.
[10] Y. Jin, C. Zhao, Y. Wang, Q. Jiang, C. Ji, M. Jia, Ionics **2017**, 23, 3249-3254.
[11] S. Hou, X. Xu, M. Wang, Y. Xu, T. Lu, Y. Yao, L. Pan, Journal of Materials Chemistry A **2017**, 5, 19054-19061.
[12] M. Cheng, H. Fan, Y. Xu, R. Wang, X. Zhang, Nanoscale **2017**, 9, 14162-14171.
[13] X. Chen, M. Cheng, D. Chen, R. Wang, ACS Applied Materials & Interfaces **2016**, 8, 3892-3900.
[14] Y. Jin, C. Zhao, Q. Jiang, C. Ji, Applied Surface Science **2018**, 450, 170-179.
[15] A. M. Elshahawy, C. Guan, X. Li, H. Zhang, Y. Hu, H. Wu, S. J. Pennycook, J. Wang, Nano Energy **2017**, 39, 162-171.
[16] S. Xie, J. Gou, Journal of Alloys and Compounds **2017**, 713, 10-17.
[17] B. Li, Y. Shi, K. Huang, M. Zhao, J. Qiu, H. Xue, H. Pang, Small **2018**, 14, 1703811.
[18] G.-C. Li, P.-F. Liu, R. Liu, M. Liu, K. Tao, S.-R. Zhu, M.-K. Wu, F.-Y. Yi, L. Han, Dalton Transactions **2016**, 45, 13311-13316.
[19] H. Hu, B. Guan, B. Xia, X. W. Lou, Journal of the American Chemical Society **2015**, 137, 5590-5595.
[20] L. Quan, T. Liu, M. Yi, Q. Chen, D. Cai, H. Zhan, Electrochimica Acta **2018**, 281, 109-116.
[21] L. Hou, Y. Shi, C. Wu, Y. Zhang, Y. Ma, X. Sun, J. Sun, X. Zhang, C. Yuan, Advanced Functional Materials **2018**, 28, 1705921.
[22] P. Zhang, B. Y. Guan, L. Yu, X. W. Lou, Angewandte Chemie International Edition **2017**, 56, 7141-7145.
[23] G. Yilmaz, K. M. Yam, C. Zhang, H. J. Fan, G. W. Ho, Advanced Materials **2017**, 29, 1606814.
[24] Y. M. Chen, Z. Li, X. W. Lou, Angewandte Chemie International Edition **2015**, 54, 10521-10524.
[25] B. Y. Guan, L. Yu, X. Wang, S. Song, X. W. Lou, Advanced Materials **2017**, 29, 1605051.
[26] S. Dai, B. Zhao, C. Qu, D. Chen, D. Dang, B. Song, B. M. deGlee, J. Fu, C. Hu, C.-P. Wong, M. Liu, Nano Energy **2017**, 33, 522-531.
[27] B. Zhao, L. Zhang, Q. Zhang, D. Chen, Y. Cheng, X. Deng, Y. Chen, R. Murphy, X. Xiong, B. Song, C.-P. Wong, M.-S. Wang, M. Liu, Advanced Energy Materials **2018**, 8, 1702247.
